# The prediction of late-onset preeclampsia: Results from a longitudinal proteomics study

**DOI:** 10.1371/journal.pone.0181468

**Published:** 2017-07-24

**Authors:** Offer Erez, Roberto Romero, Eli Maymon, Piya Chaemsaithong, Bogdan Done, Percy Pacora, Bogdan Panaitescu, Tinnakorn Chaiworapongsa, Sonia S. Hassan, Adi L. Tarca

**Affiliations:** 1 Perinatology Research Branch, Program for Perinatal Research and Obstetrics, Division of Intramural Research, *Eunice Kennedy Shriver* National Institute of Child Health and Human Development, National Institutes of Health, U.S. Department of Health and Human Services, Bethesda, Maryland, and Detroit, Michigan, United States of America; 2 Department of Obstetrics and Gynecology, Wayne State University School of Medicine, Detroit, Michigan, United States of America; 3 Maternity Department “D” and Obstetrical Day Care Center, Division of Obstetrics and Gynecology, Soroka University Medical Center, School of Medicine, Faculty of Heath Sciences, Ben Gurion University of the Negev, Beer Sheva, Israel; 4 Department of Obstetrics and Gynecology, University of Michigan, Ann Arbor, Michigan, United States of America; 5 Department of Epidemiology and Biostatistics, Michigan State University, East Lansing, Michigan, United States of America; 6 Center for Molecular Medicine and Genetics, Wayne State University, Detroit, Michigan, United States of America; 7 Department of Computer Science, Wayne State University College of Engineering, Detroit, Michigan, United States of America; Hungarian Academy of Sciences, HUNGARY

## Abstract

**Background:**

Late-onset preeclampsia is the most prevalent phenotype of this syndrome; nevertheless, only a few biomarkers for its early diagnosis have been reported. We sought to correct this deficiency using a high through-put proteomic platform.

**Methods:**

A case-control longitudinal study was conducted, including 90 patients with normal pregnancies and 76 patients with late-onset preeclampsia (diagnosed at ≥34 weeks of gestation). Maternal plasma samples were collected throughout gestation (normal pregnancy: 2–6 samples per patient, median of 2; late-onset preeclampsia: 2–6, median of 5). The abundance of 1,125 proteins was measured using an aptamers-based proteomics technique. Protein abundance in normal pregnancies was modeled using linear mixed-effects models to estimate mean abundance as a function of gestational age. Data was then expressed as multiples of-the-mean (MoM) values in normal pregnancies. Multi-marker prediction models were built using data from one of five gestational age intervals (8–16, 16.1–22, 22.1–28, 28.1–32, 32.1–36 weeks of gestation). The predictive performance of the best combination of proteins was compared to placental growth factor (PIGF) using bootstrap.

**Results:**

1) At 8–16 weeks of gestation, the best prediction model included only one protein, matrix metalloproteinase 7 (MMP-7), that had a sensitivity of 69% at a false positive rate (FPR) of 20% (AUC = 0.76); 2) at 16.1–22 weeks of gestation, MMP-7 was the single best predictor of late-onset preeclampsia with a sensitivity of 70% at a FPR of 20% (AUC = 0.82); 3) after 22 weeks of gestation, PlGF was the best predictor of late-onset preeclampsia, identifying 1/3 to 1/2 of the patients destined to develop this syndrome (FPR = 20%); 4) 36 proteins were associated with late-onset preeclampsia in at least one interval of gestation (after adjustment for covariates); 5) several biological processes, such as *positive regulation of vascular endothelial growth factor receptor signaling pathway*, were perturbed; and 6) from 22.1 weeks of gestation onward, the set of proteins most predictive of severe preeclampsia was different from the set most predictive of the mild form of this syndrome.

**Conclusions:**

Elevated MMP-7 early in gestation (8–22 weeks) and low PlGF later in gestation (after 22 weeks) are the strongest predictors for the subsequent development of late-onset preeclampsia, suggesting that the optimal identification of patients at risk may involve a two-step diagnostic process.

## Introduction

Preeclampsia, a frequent complication of pregnancy that affects 5%-8% of all gestations [1–3], is a leading cause of maternal [2–13] and perinatal morbidity and mortality [4,14–16]. Over the last decade, it has become clear that preeclampsia is not a single disorder but a syndrome with many etiologies [17–21], such as abnormal placentation [19,22–24], utero-placental ischemia [20,25–29], vascular disorders of the placenta [30–32], insulin resistance [33–40], systemic maternal inflammation [41–44], endothelial dysfunction [45–50], and imbalance of angiogenic and anti-angiogenic proteins [26,51–76].

A major advance in the classification of preeclampsia was its subdivision into early- (<34 weeks of gestation) and late-onset variants [77–79]. Although other cut-off values between early- and late-onset diseases have been suggested, such as 32 and 36 weeks of gestation [80–82], the value of 34 weeks remains the most commonly used [83–85], presumably because the rate of neonatal morbidity declines considerably after 34 weeks [86–88].

Late-onset preeclampsia is more common than its early-onset variant [83,89] and accounts for 90% of cases [12] and a substantial fraction of maternal complications [12]. For example, in a study from South Africa [89], late-onset preeclampsia accounted for 30% of severe maternal complications, 13% of eclampsia, and 1.9% of fetal deaths [89].

Early-onset preeclampsia is likely caused by a disorder of deep placentation in which there is a failure of physiologic transformation of the spiral arteries, a small placenta with histologic features of maternal vascular underperfusion [90–94], fetal growth restriction or small for gestational age [95–98], and abnormal Doppler velocimetry of umbilical and uterine arteries [99–104]; it frequently requires preterm delivery for maternal and/or fetal indications [12]. By contrast, late-onset preeclampsia seems to be the manifestation of a mismatch between the metabolic demands of the growing fetus close to term and maternal supply [77–79]: the placenta has fewer lesions of maternal vascular underperfusion [90–93] and abnormalities of umbilical/uterine artery Doppler velocimetry [79]. Consistent with these findings, in most cases of late-onset preeclampsia, neonates are appropriate or large for gestational age [105–108].

Maternal hemodynamic status differs in patients with early- and late-onset preeclampsia [79]. These differences can be identified as early as 24 weeks of gestation. Those who develop late-onset preeclampsia have increased cardiac output and relatively unchanged total vascular resistance [79], whereas patients with early-onset preeclampsia have lower cardiac output and relatively increased vascular resistance.

The angiogenic/anti-angiogenic imbalance is milder in late-onset rather than in early-onset preeclampsia [58,59,72,80,82,83,109–112]: indeed, it has been suggested that angiogenic/anti-angiogenic factors can be used as biomarkers to identify patients destined to develop early preeclampsia. These include placental growth factor (PlGF), soluble vascular endothelial growth factor receptor 1, endoglin, and their ratios [55,58,65,82,109,113–117]. Prediction models have also been developed that combine maternal blood pressure, uterine artery Doppler, PlGF [99,101,118–124], pregnancy-associated plasma protein A, and inhibin-A [125]. One such model has been used to identify nulliparous women at risk for early preeclampsia [121] and to design a randomized clinical trial of aspirin in early pregnancy to prevent early-onset preeclampsia [126]. However, the predictive performance (especially in early pregnancy) of models, including angiogenic/anti-angiogenic factors, for the identification of women destined to develop late-onset preeclampsia was lower than those for early-onset disease [82,114,118,119].

New technology, not based on antigen-antibody reactions, has been developed to increase the number of proteins that can be detected simultaneously with a high degree of sensitivity and dynamic range [127,128]. This aptamer-based method uses single-strand DNA or RNA molecules that bind to proteins, peptides, or other pre-defined molecules with high affinity and specificity. The use of aptamer technology for the discovery of biomarkers for cardiovascular disease [129] and other medical conditions [130–135] has recently been reported, and we have previously reported changes in the maternal plasma proteome as a function of gestational age [128]. Therefore, we used this high through-put proteomic platform to identify proteins that can serve as biomarkers for the identification of patients who subsequently develop late-onset preeclampsia.

## Materials and methods

### Study design

A retrospective nested case-control study was designed to include 90 patients with normal pregnancies (controls) and 76 patients with late-onset preeclampsia defined as preeclampsia diagnosed at ≥34 weeks of gestation). Patients were enrolled between February 2007 and Dec 2013 as part of a longitudinal cohort study conducted at the Center for Advanced Obstetrical Care and Research of the Perinatology Research Branch, NICHD/NIH/DHHS, the Detroit Medical Center and Wayne State University. Plasma samples were collected at the time of each prenatal visit scheduled at four-week intervals from the first or early second trimester until delivery. Each patient had at least two samples collected during the following gestational age intervals: 8-<16 weeks, 16-<24 weeks, 24-<28 weeks, 28-<32 weeks, 32-<37 weeks and >37 weeks. The median number (range) of samples per patient was 5(2–6) for cases and 2 (2–6) for controls. All patients provided written informed consent, and the use of biological specimens, as well as clinical and ultrasound data for research purposes, were approved by the Wayne State University Human Investigation Committee and the Institutional Review Board of NICHD.

### Clinical definitions

Preeclampsia was defined as new-onset hypertension that developed after 20 weeks of gestation (systolic or diastolic blood pressure ≥140 and/or ≥90 mm Hg, respectively, measured on at least two occasions, 4 hours to 1 week apart) and proteinuria (≥300 mg in a 24-hour urine collection, or two random urine specimens obtained 4 hours to 1 week apart containing ≥1+ by dipstick or one dipstick demonstrating ≥2+ protein) [83,136].

Early-onset preeclampsia was defined as preeclampsia diagnosed before 34 weeks [83]. Late-onset preeclampsia was defined as preeclampsia diagnosed at or after 34 weeks of gestation.

Mild preeclampsia was diagnosed as preeclampsia with systolic blood pressure < 160 mmHg, or diastolic blood pressure < 110 mmHg, platelet count ≥ 100,000 per mm^3^, non-elevated liver enzymes, absence of renal insufficiency, pulmonary edema, cyanosis, new-onset cerebral/visual disturbances, and/or right upper quadrant or epigastric pain [20,85].

Severe preeclampsia was diagnosed as preeclampsia with systolic blood pressure ≥ 160 mmHg, or diastolic blood pressure ≥ 110 mmHg, platelet count < 100,000 per mm^3^, elevated liver enzymes, renal insufficiency, pulmonary edema or cyanosis, new-onset cerebral/visual disturbances, and/or right upper quadrant or epigastric pain [20,85].

Body mass index (BMI) was calculated as follows: BMI = [weight (in pounds) × 703)/height^2^ (in inches)]. Obesity was defined as BMI ≥ 30 kg/m^2^ [137].

### Proteomic analysis

Maternal plasma protein abundance was determined using the SOMAmer (Slow Off-rate Modified Aptamers) platform and its reagents that allowed the abundance of 1,125 proteins to be profiled [138–140]. Proteomics profiling services were provided by Somalogic, Inc. (Boulder, CO, USA) in December 2014.

The serum samples were diluted and then incubated with the respective SOMAmer mixes pre-immobilized onto streptavidin-coated beads. The beads were washed in order to remove all non-specifically bound proteins and other matrix constituents. Proteins that remained bound to their cognate SOMAmer reagents were tagged using an NHS-biotin reagent. After the labeling reaction, the beads were exposed to an anionic competitor solution to prevent non-specific interactions from reforming after disruption.

Using this approach, pure cognate-SOMAmer complexes and unbound (free) SOMAmer reagents are released from the streptavidin beads using ultraviolet light that cleaves the photo-cleavable linker used to quantitate proteins. The photo-cleavage eluate, which contains all SOMAmer reagents (some bound to a biotin-labeled protein and some free), was separated from the beads and then incubated with a second streptavidin-coated bead that binds the biotin-labeled proteins and the biotin-labeled protein-SOMAmer complexes. The free SOMAmer reagents were then removed using subsequent washing steps. In the final elution step, protein-bound SOMAmer reagents were released from their cognate proteins using denaturing conditions. These SOMAmer reagents were then quantified by hybridization to custom DNA microarrays. The Cyanine-3 signal from the SOMAmer reagent was detected on microarrays and used for quantification [138–140].

### Statistical analysis

#### Demographics data analysis

Clinical characteristics of the patient population were summarized as median and inter-quartile ranges (IQR) for continuous variables or as percentages for categorical variables. Comparisons of the demographics variables between groups were performed using a Fisher’s exact test (for binary variables) and the Wilcoxon rank-sum test for continuous variables.

#### Data transformation

The raw protein abundance data consisted of relative fluorescence units obtained from scanning the microarrays with a laser scanner. A sample-by-sample adjustment of the overall signal within a single plate (85 samples processed per plate/run) was performed in three steps: Hybridization Control Normalization, Median Signal Normalization, and Calibration, using the manufacturer’s protocol. Outlier protein abundance values above 2 *×* the 98^th^ percentile of all samples, were replaced with 2 *×* the 98^th^ percentile of all samples (data thresholding) (See S1 File for the protein abundance data after the thresholding step). Protein abundance was then log (base 2) transformed to improve normality. Linear mixed-effects models with cubic splines (number of knots = 3) were used to model protein abundance in controls as a function of gestational age using the *lme4* package [141] under the R statistical language and environment (www.r-project.org). Data for all samples was then expressed as multiples-of-the-mean (MoM) values for the corresponding gestational age in normal pregnancies.

#### Development of multi-marker prediction models

The goal of this analysis was to develop parsimonious, accurate prediction models by using protein abundance in each gestational age interval separately (8–16, 16.1–22, 22.1–28, 28.1–32, 32.1–36 weeks of gestation) applying predictive modeling techniques for omics data that we previously reported [142–144]. Log (base 2) MoM values for one protein at a time were used to fit a Linear Discriminant Analysis (LDA) model, and compute, by leave-one-out cross-validation (LOOCV), a classification performance measure for each protein. This performance measure was the partial Area Under the Receiver Operating Characteristic (ROC) curve (pAUC) using a cut-off of 0.5 false positive rate. The use of a partial as opposed to the full area under the ROC curve was chosen to emphasize the need to find proteins that have high sensitivity at low false positive rates. Further, proteins that did not change at least 10% in average abundance between the groups were removed from the analysis. Then, LDA models were fit using increasing sets of up to 5 of the top proteins ranked by the pAUC. To enforce model parsimony, the inclusion of each additional protein was conditioned on the increase of 0.01 units in a pAUC statistic. Classification performance indices [AUC, sensitivity, specificity, positive and negative predictive values, likelihood ratio (+) and (-)] were obtained for the best combinations of markers in each interval by LOOCV. While this accounts for biases due to over-fitting of the data for a given set of selected proteins, it does not account for the fact that those proteins were selected from a large pool of candidate predictors. Therefore, classification performance indices were also obtained using bootstrap. With this approach, after data transformation into MoM, patients (both cases and controls) were selected with replacement. All analysis steps involved in the prediction model development (including selection of predictor proteins) were performed using only data from the selected patients (training set) and prediction performance was calculated by applying the resulting model on the patients left out (test set). Averages of 100 such bootstrap iterations are reported and the discussion of results is based primarily on these performance estimates since they are considered most robust.

#### Differential abundance analysis

Since the classifier development pipeline described above is focused on finding the most accurate, parsimonious set of proteins that predict late-onset preeclampsia, it will not necessarily retain all proteins showing evidence of differential abundance. Therefore, a complementary analysis was performed to test for differences between mean log (base 2) MoM values between cases and controls at each gestational age interval. Linear models with coefficient significance evaluated via moderated t-tests were implemented using the *limma* package [145] of Bioconductor repository [146]. With this procedure, standard deviation estimates of log2 MoM values for each protein are shrunk toward a common (pooled) value to improve robustness. Significance was inferred based on the false discovery rate adjusted p-value (q-value) <0.25 and fold-change in abundance >1.1 fold after adjusting for BMI, smoking status, maternal age, and parity.

#### Gene ontology enrichment analysis

Proteins selected as differentially abundant between late-onset preeclampsia and normal pregnancy in each interval of gestation were mapped to Entrez gene identifiers [147] based on Somalogic, Inc., annotation, and then to gene ontology [148]. Biological processes over-represented among the proteins that changed with late-onset preeclampsia were identified using a Fisher’s exact test. Gene ontology terms with three or more hits and q-values <0.1 were considered significantly enriched.

## Results

### Clinical characteristics of the study population

Women with late-onset preeclampsia had a lower median gestational age at delivery (p<0.001) and a higher median maternal BMI (p = 0.03) than the controls. Thirty-seven percent (28/76) of cases had severe preeclampsia and 63% (48/76) had mild preeclampsia. Median gestational age at delivery was lower both in patients who had mild preeclampsia and in those who had severe preeclampsia than in the controls (p<0.001), but the median maternal BMI was higher than the controls only in patients who had severe preeclampsia (p = 0.01) (Table 1).

**Table 1:** Demographic characteristics of the study population.

Characteristic	Normal (n = 90)	Late PE (n = 76)	Mild Late PE (n = 48)	Severe Late PE (n = 28)
Gestational age at delivery (weeks)	39.4 (39.0–40.4)	38.7 (37.7–39.4) [p<0.001]	38.8 (37.7–39.4) [p<0.001]	38.6 (37.7–39.5) [p<0.001]
BMI (kg/m^2^)	26.5 (22.8–33.2)	30.0 (24.8–36.2) [p = 0.03]	28.4 (24.0–33.0) [p = 0.23]	32.5 (27.2–38.7) [p = 0.01]
Maternal age (years)	24 (21.0–27.8)	22 (20.0–29.0) [p = 0.45]	22.5 (20.0–28.3) [p = 0.57]	22 (19.0–29.3) [p = 0.5]
Smoking	18 (20%)	13 (17.1%) [p = 0.7]	9 (18.8%) [p = 1]	4 (14.2%) [p = 0.6]
Nulliparity	26 (28.9%)	32 (42.1%) [p = 0.1]	22 (45.8%) [p = 0.06]	10 (35.7%) [p = 0.5]

Data is presented as median (interquartile range) or as percentage (n); BMI, body mass index. P values are given for the comparison to the normal pregnancy group.

### Proteomic prediction models for late-onset preeclampsia prior to diagnosis

**Fig 1:**
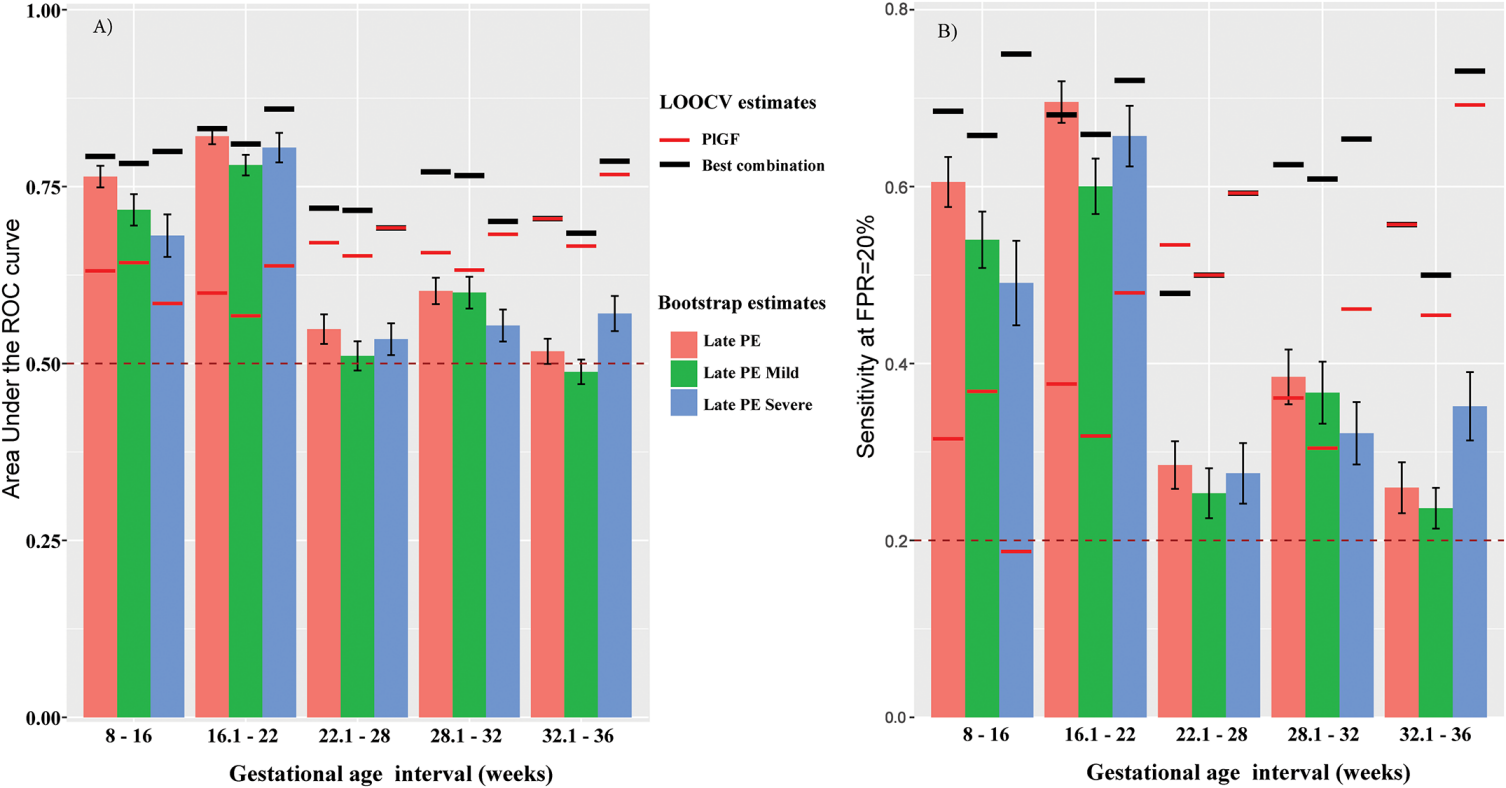
Prediction of late-onset preeclampsia using multi-protein markers. The area under the Receiver Operating Characteristic (ROC) curve (A) and the sensitivity at a 20% false positive rate (FPR) (B) are shown. Estimates obtained by leave-one-out cross-validation (LOOCV) for the best combination of markers (Table 2) is shown as horizontal black segments for late preeclampsia (PE) as well as for mild and severe phenotypes of the disease. The LOOCV performance of PlGF alone is shown with red horizontal segments. The bars in this figure represent average (whiskers are with 95% confidence intervals [CI]) prediction performance obtained from 100 bootstrap iterations. For prediction of severe preeclampsia at 28.1–32 weeks and for overall preeclampsia at 32.1–36 weeks, PlGF was the only predictor selected in the final model.

Fig 1 depicts a summary of the LOOCV (black segments for best combination of markers; red segments, PlGF alone) and bootstrap (bars with 95% CI) based performance estimates for the prediction of late preeclampsia. The bootstrap estimates of AUC (Fig 1A) and sensitivity at a 20% false positive rate (FPR) (Fig 1B) achieved by the best combinations of proteins were significantly higher than those of PlGF in the first two gestational age intervals (8–16 and 16.1–22 weeks) (bars higher than red line segments).

**Fig 2:**
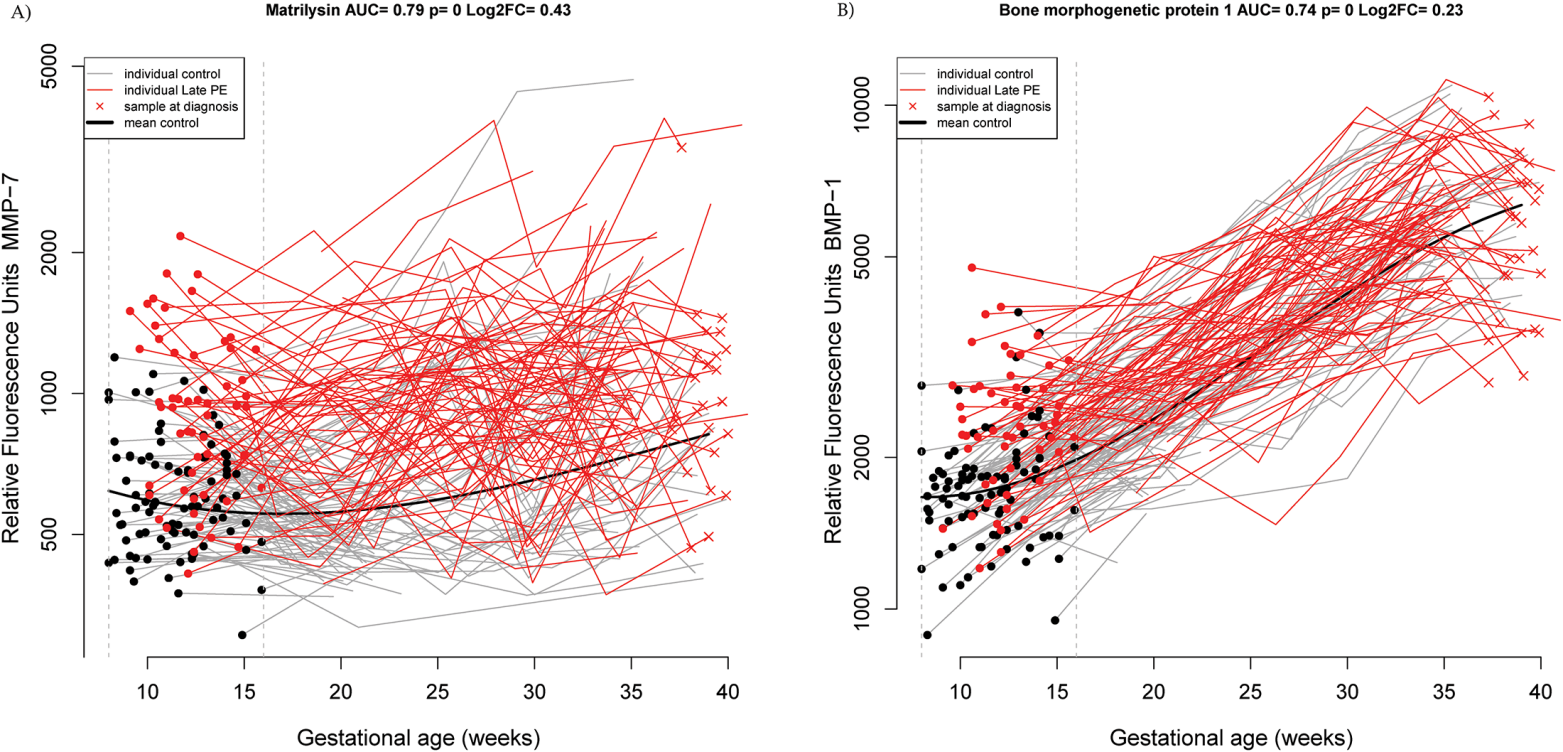
Longitudinal maternal plasma MMP-7 (A) and BMP−1 (B) abundance in normal pregnancy and late-onset preeclampsia cases, highlighting differences in the 8–16 weeks interval. Each line corresponds to one patient (grey = normal pregnancy, red = late-onset preeclampsia). Individual dots correspond to samples in the current gestational age interval (see vertical interrupted lines). The thick black line represents the mean value in normal pregnancy estimated by linear mixed-effects models. AUC is the area under the Receiver Operating Characteristic (ROC) curve of the protein using data in the current interval only; p is the nominal significance p-value comparing mean MoM values between groups with a moderated t-test. Log_2_FC is the log (base 2) of the fold-change between cases and controls, with negative values denoting lower MoM values in cases than in controls.

At **8–16 weeks** of gestation, the best combination of proteins included only matrix metalloproteinase 7 (MMP-7) that had a sensitivity of 69% at a FPR of 20% (black segment on top of the red bar at 8–16 weeks, Fig 1B) and 57% at a FPR of 10% (AUC = 0.79; see black segment at 8–16 weeks, Fig 1A and Table 2). Individual patient longitudinal MMP-7 profiles are depicted in Fig 2A, highlighting the differences in the samples taken between 8–16 weeks of gestation. When random sets of cases and controls were selected with replacement, and the entire procedure to build the classification model was repeated, MMP-7 was chosen as the best predictor in 88 of the 100 bootstrap trials and the typical (mean) AUC of the prediction model was 0.76 (see Table 3, and red bar at 8–16 weeks Fig 1A). The consistency of bootstrap-based (AUC = 0.76) and final model estimates (AUC = 0.79) of prediction performance suggest minimal to no data over-fitting. The second most frequently selected predictor protein (23/100 iterations) either by itself or in combination with other proteins was BMP-1 (AUC = 0.74) (see Fig 2B).

**Table 2:** Summary of prediction performance of multi-protein prediction models for late-onset, mild late-onset, and severe late preeclampsia.

Outcome	Sample GA (weeks)	N (Ctrls. /Cases)	Model Predictors	AUC	Sens. FPR = 20%	Sens.	Spec.	PPV	NPV	LR(+)	LR(-)
Late PE	8–16	89/54	MMP-7	0.79	0.69	0.57	0.90	0.78	0.78	5.7	0.47
Late PE	16.1–22	87/69	MMP-7	0.83	0.68	0.62	0.89	0.81	0.75	5.4	0.43
Late PE	22.1–28	43/73	RAN+METAP1	0.72	0.48	0.23	0.86	0.74	0.40	1.7	0.89
Late PE	28.1–32	40/72	RAN+CAMK2A+TF	0.77	0.63	0.50	0.85	0.86	0.49	3.3	0.59
Late PE	32.1–36	39/70	PlGF	0.70	0.56	0.26	0.87	0.78	0.40	2.0	0.85
Mild	8–16	89/38	MMP-7 + Phosphoglycerate mutase 1	0.78	0.66	0.47	0.89	0.64	0.80	4.2	0.59
Mild	16.1–22	87/44	MMP-7	0.81	0.66	0.59	0.90	0.74	0.81	5.7	0.46
Mild	22.1–28	43/46	METAP1+RAN	0.72	0.50	0.17	0.88	0.62	0.50	1.5	0.93
Mild	28.1–32	40/46	TF+RAN+FER	0.77	0.61	0.41	0.85	0.76	0.56	2.8	0.69
Mild	32.1–36	39/44	TF	0.68	0.50	0.43	0.90	0.83	0.58	4.2	0.63
Severe	8–16	89/16	MMP-7	0.80	0.75	0.63	0.89	0.50	0.93	5.6	0.42
Severe	16.1–22	87/25	MMP-7	0.86	0.72	0.68	0.90	0.65	0.91	6.6	0.36
Severe	22.1–28	43/27	PlGF	0.69	0.59	0.52	0.88	0.74	0.75	4.5	0.54
Severe	28.1–32	40/26	PTP-1B	0.70	0.65	0.42	0.93	0.79	0.71	5.6	0.62
Severe	32.1–36	39/26	PlGF + Histone H2A.z	0.79	0.73	0.50	0.82	0.65	0.71	2.8	0.61

GA: gestational age; AUC: area under the receiver operating characteristic curve; FPR: false-positive rate; Sens. Sensitivity, Spec. Specificity, PPV: Positive Predicted Value, NPV: Negative Predicted Value, LR: Likelihood Ratio (LR).

**Table 3:** Summary of prediction performance for late-onset preeclampsia evaluated by bootstrap.

Outcome	Sample GA (weeks)	AUC	Sens.	Spec.	Predictor Symbols (# of inclusions in the best combination)
Late PE	8–16	0.76	0.61	0.80	MMP-7(88); BMP-1(23); CDK8/cyclin C(12)
Late PE	16.1–22	0.82	0.70	0.80	MMP-7(94); HMG-1(18); gpIIbIIIa(17); Integrin aVb5(10)
Late PE	22.1–28	0.55	0.29	0.80	PlGF(24); METAP1(16); MMP-7(15); RAN(12)
Late PE	28.1–32	0.60	0.38	0.80	RAN(44); CAMK2A(23); TF(14); FER(12); CAMK2D(11)
Late PE	32.1–36	0.52	0.26	0.79	Cathepsin B(21); PlGF(20); BMPER(11)
Mild	8–16	0.72	0.54	0.80	MMP-7(74); Phosphoglycerate mutase 1(17); BMP-1(12); CDK8/cyclin C(10)
Mild	16.1–22	0.78	0.60	0.80	MMP-7(84); gpIIbIIIa(27); HMG-1(12)
Mild	22.1–28	0.51	0.25	0.79	RAN(15); PlGF(13); Cathepsin B(10)
Mild	28.1–32	0.60	0.37	0.80	RAN(41); TF(30); FER(20)
Mild	32.1–36	0.49	0.24	0.79	Cathepsin B(16); TF(13)
Severe	8–16	0.68	0.49	0.80	MMP-7(52); BMP-1(23); PPID(10)
Severe	16.1–22	0.81	0.66	0.80	MMP-7(79); Integrin aVb5(34); HMG-1(18)
Severe	22.1–28	0.53	0.28	0.79	PlGF(20)
Severe	28.1–32	0.55	0.32	0.80	PTP-1B(18)
Severe	32.1–36	0.57	0.35	0.79	PlGF(24); FCN2(10)

The number in parentheses following the name of each protein in the column labeled Predictor Symbols represents the number of bootstrap iterations for which the protein was selected in the best predictor combination. Only proteins selected 10 times or more are listed. AUC: Area under the Receiver Operating Characteristic Curve, Sens. Sensitivity, Spec. Specificity.

**Fig 3:**
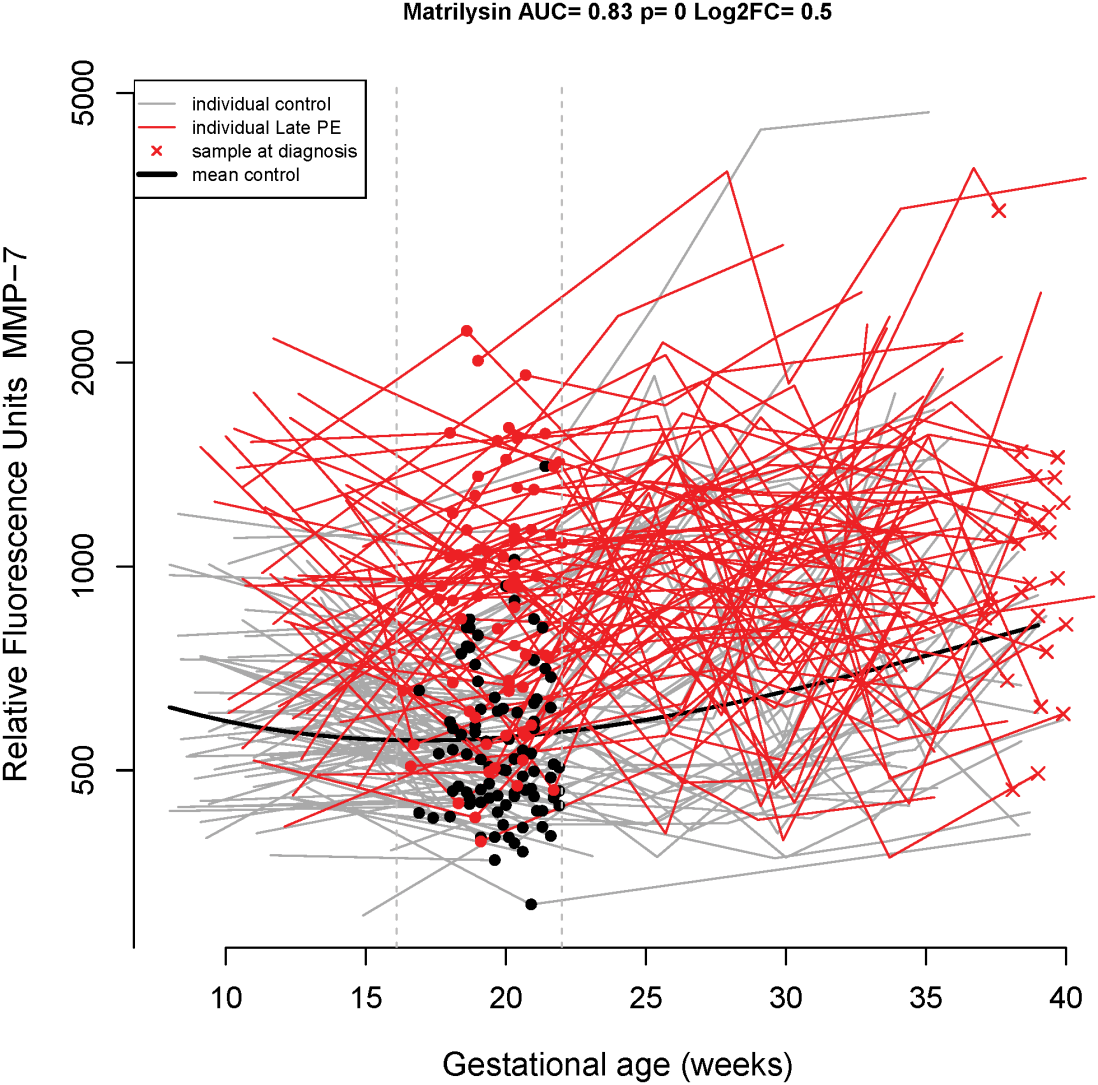
Longitudinal maternal plasma MMP-7 abundance in normal pregnancy and late-onset preeclampsia cases, highlighting differences in the 16.1–22 weeks interval. See the Fig 1 legend for more details.

At **16.1–22 weeks** of gestation, MMP-7 was again the single best predictor of late-onset preeclampsia with a sensitivity of 68% at a FPR of 20%, and 62% at a FPR of 10% (AUC = 0.83; 0.82 bootstrap estimate) (Tables 2 and 3). Longitudinal MMP-7 profiles emphasizing the differences in the samples taken between 16.1 to 22 weeks of gestation are shown in Fig 3. MMP-7 was selected in the best model of 94 of the 100 bootstrap trials with the next most frequently selected proteins HMG-1 (high-mobility group protein box-1) and gpIIbIIIa (Integrin alpha-IIb: beta-3 complex) being selected only 18 and 17 times, respectively.

**Fig 4:**
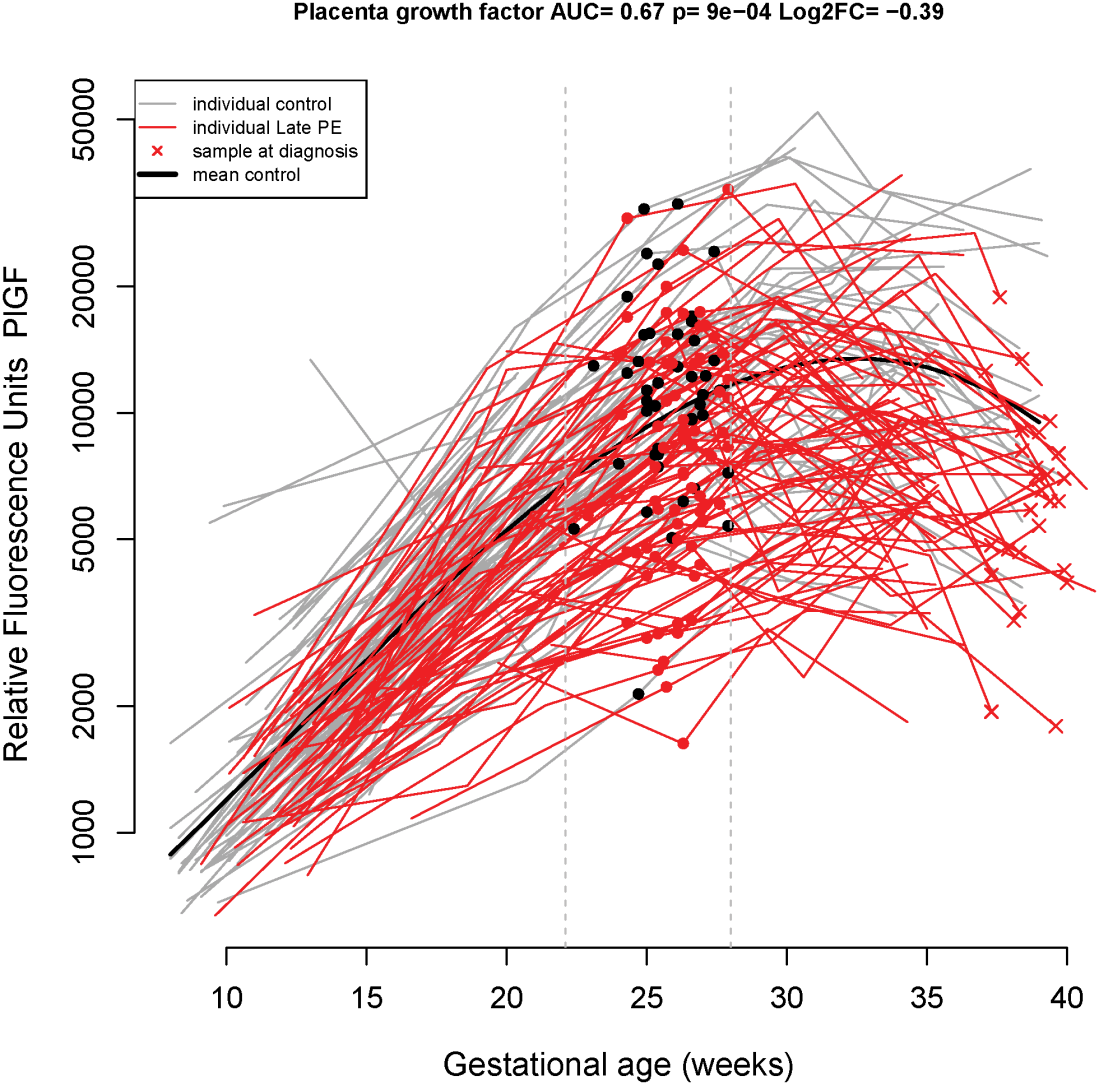
Longitudinal maternal plasma PlGF abundance in normal pregnancy and late-onset preeclampsia cases, highlighting differences in the 22.1–28 weeks interval. See the Fig 1 legend for more details.

At **22.1–28 weeks** of gestation, the proteomics profile predicted late-onset preeclampsia with a sensitivity of 48% at a FPR of 20% and with a sensitivity of 23% at FPR of 10% (AUC = 0.72). The two proteins included in the final model at this gestational age interval were RAN (RAs-related Nuclear protein, also known as GTP-binding nuclear protein Ran) and METAP1 (Methionine aminopeptidase 1). However, the bootstrap-estimated performance of combinations of proteins at this gestational age interval was substantially lower (29% sensitivity at a FPR of 20%, AUC = 0.55): PlGF (Fig 4: longitudinal profiles) was selected most frequently in the best model (24/100 times) followed by METAP1 (16/100), MMP-7(15/100) and RAN (12/100) (Table 3).

Prediction performance for late-onset preeclampsia at the 28.1–32 and 32.1–36 week intervals did not exceed the values obtained at the 8–16 and 16.1–22 week intervals, with proteins such as RAN, Calcium/calmodulin-dependent protein kinase type II alpha chain (CAMK2A), PlGF, tissue factor (TF), and Cathepsin B being among the most frequently (14 to 44 times out of 100) included as predictors in the optimal LDA prediction models for late-onset preeclampsia (Table 3).

### Prediction of late-onset preeclampsia according to its severity

When severe and mild late-onset preeclampsia cases were compared separately against the controls, the estimated prediction performance of multi-protein models was very similar to the one for overall late-onset preeclampsia (Fig 1A and 1B and Tables 2 and 3). Although for the 8–16 and 16.1–22 weeks’ intervals when MMP-7 was selected as the best model in a majority of bootstrap trials, there were differences in the top proteins included for prediction of subsequent mild as opposed to severe late-onset preeclampsia (Table 3). PlGF, PTP-1B (Tyrosine-protein phosphatase non-receptor type 1), and FCN2 (Ficolin-2) were the most frequently selected to predict severe preeclampsia (10-24/100 times) while RAN, TF, FER, and Cathepsin B were the most frequently selected in the best combinations of predictors of mild late-onset preeclampsia (Table 3). Since combinations of proteins did not perform any better than PlGF alone, we describe only the prediction performance indices for PlGF in the intervals from 22.1–36 weeks of gestation (see red line segments in Fig 1): at 22.1–28 weeks, the sensitivity of PlGF was 53% (FPR = 20%) for overall late-onset preeclampsia (50% for mild and 59% for severe preeclampsia) (Fig 1B); at 28.1–32 weeks, the sensitivity of PlGF was 36% (FPR = 20%) for overall late-onset preeclampsia (30% for mild and 46% for severe preeclampsia) (Fig 1B). At 32.1–36 weeks, the sensitivity of PlGF was 56% (FPR = 20%) for overall late-onset preeclampsia (45% for mild and 69% for severe preeclampsia) (Fig 1B).

### Differential protein abundance summary

**Fig 5:**
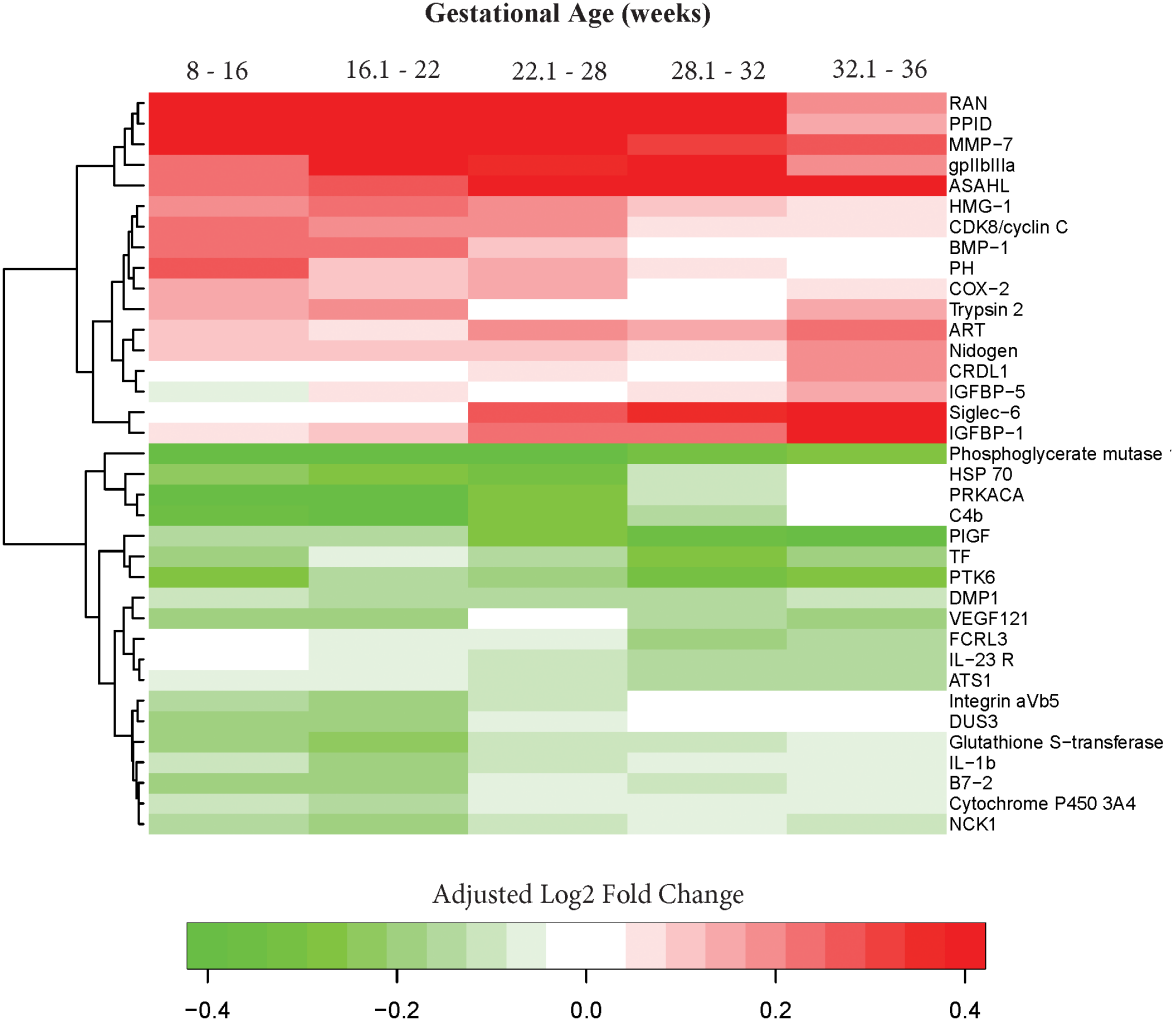
A summary of differential protein abundance between late-onset preeclampsia and normal pregnancy throughout gestation. The value shown using a color scheme represents the log_2_ fold-change in MoM values between cases and controls (green = lower, red = higher mean MoM in cases than in controls). Absolute fold-changes >1.3 (absolute log2 fold change >0.4) were re-set to 1.3 in order to enhance visualization of the data.

In addition to the few proteins that were included in the parsimonious models predictive of late-onset preeclampsia at different gestational age intervals (Table 2), 36 additional proteins showed evidence for differential abundance after adjusting for BMI, smoking status, maternal age, and parity (q-value<0.25 and fold change >1.1) in at least one interval of gestation. Table 4 shows the linear fold-changes in the MoM values between late-onset preeclampsia and the control groups, as well as the nominal and FDR adjusted p-values (q-values) for each gestational age interval. The heatmap summarizes the differential abundance patterns across all gestational age intervals considered (Fig 5 and Table 4). Notably, the abundance of MMP-7, CDK8/cyclin C (Cyclin-dependent kinase 8:Cyclin-C complex), PPID (Peptidylprolyl isomerase D), and RAN were higher while the abundance of HSP70 (Heat shock 70 kDa protein 1A/1B) was lower in cases compared to the controls in the first three gestational age intervals (8–16, 16.1–22, 22.1–28 weeks).

**Table 4:** Summary of differential abundance analysis between late-onset preeclampsia and normal pregnancy in five intervals of gestation.

	8–16 (weeks)		16.1–22 (weeks)		22.1–28 (weeks)		28.1–32 (weeks)		32.1–36 (weeks)	
SYMBOL	FC	Sig.	FC	Sig.	FC	Sig.	FC	Sig.	FC	Sig.
PPID	1.4	**Yes**	1.4	**Yes**	1.6	**Yes**	1.3	No	1.1	No
RAN	1.4	**Yes**	1.4	**Yes**	1.6	**Yes**	1.5	No	1.1	No
MMP-7	1.4	**Yes**	1.4	**Yes**	1.3	**Yes**	1.2	No	1.2	No
CDK8/cyclin C	1.2	**Yes**	1.1	**Yes**	1.1	**Yes**	1.1	No	1.0	No
HSP 70	-1.2	**Yes**	-1.2	**Yes**	-1.2	**Yes**	-1.1	No	1.0	No
BMP-1	1.2	**Yes**	1.2	**Yes**	1.1	No	1.0	No	-1.0	No
HMG-1	1.1	**Yes**	1.2	**Yes**	1.1	No	1.1	No	1.0	No
Glutathione S-transferase Pi	-1.1	**Yes**	-1.2	**Yes**	-1.1	No	-1.1	No	-1.0	No
C4b	-1.3	**Yes**	-1.3	**Yes**	-1.2	No	-1.1	No	-1.0	No
PH	1.2	**Yes**	1.1	No	1.1	No	1.0	No	1.0	No
COX-2	1.1	**Yes**	1.1	No	1.1	No	1.0	No	1.0	No
Phosphoglycerate mutase 1	-1.8	**Yes**	-1.4	No	-1.4	No	-1.2	No	-1.2	No
gpIIbIIIa	1.2	No	1.5	**Yes**	1.3	No	1.3	No	1.1	No
Trypsin 2	1.1	No	1.1	**Yes**	1.0	No	-1.0	No	1.1	No
Cytochrome P450 3A4	-1.1	No	-1.1	**Yes**	-1.0	No	-1.1	No	-1.0	No
IL-1b	-1.1	No	-1.1	**Yes**	-1.1	No	-1.0	No	-1.0	No
DMP1	-1.1	No	-1.1	**Yes**	-1.1	No	-1.1	No	-1.1	No
Integrin aVb5	-1.1	No	-1.1	**Yes**	-1.1	No	-1.0	No	1.0	No
NCK1	-1.1	No	-1.1	**Yes**	-1.1	No	-1.1	No	-1.1	No
B7-2	-1.1	No	-1.1	**Yes**	-1.0	No	-1.1	No	-1.0	No
VEGF121	-1.1	No	-1.1	**Yes**	-1.0	No	-1.1	No	-1.1	No
DUS3	-1.1	No	-1.1	**Yes**	-1.0	No	-1.0	No	1.0	No
PRKACA	-1.3	No	-1.3	**Yes**	-1.2	No	-1.1	No	-1.0	No
ASAHL	1.2	No	1.2	No	1.4	No	1.4	**Yes**	1.4	**Yes**
TF	-1.1	No	-1.0	No	-1.1	No	-1.2	**Yes**	-1.1	No
PTK6	-1.2	No	-1.1	No	-1.1	No	-1.2	**Yes**	-1.2	No
Nidogen	1.1	No	1.1	No	1.1	No	1.1	No	1.1	**Yes**
ART	1.1	No	1.1	No	1.1	No	1.1	No	1.2	**Yes**
IGFBP-1	1.1	No	1.1	No	1.2	No	1.2	No	1.3	**Yes**
Siglec-6	1.0	No	1.0	No	1.2	No	1.3	No	1.5	**Yes**
FCRL3	1.0	No	-1.0	No	-1.0	No	-1.1	No	-1.1	**Yes**
CRDL1	1.0	No	1.0	No	1.0	No	1.0	No	1.1	**Yes**
IL-23 R	-1.0	No	-1.0	No	-1.1	No	-1.1	No	-1.1	**Yes**
IGFBP-5	-1.0	No	1.0	No	1.0	No	1.0	No	1.1	**Yes**
ATS1	-1.0	No	-1.1	No	-1.1	No	-1.1	No	-1.1	**Yes**
PlGF	-1.1	No	-1.1	No	-1.2	No	-1.3	No	-1.3	**Yes**

Thirty-six proteins that were significant (Sig.) (q<0.25 and fold change >1.1) in at least one interval are shown. Adjustment was performed for BMI, maternal age, parity and smoking. FC: linear fold change, with negative values denoting lower while positive values denoting higher level in cases than in controls. See S2 File for full names of proteins, p-values and q-values as well as for which proteins change in abundance during gestation.

Of the 36 proteins associated with late-onset preeclampsia in at least one gestational age interval, 11 (31%) were among those modulated during gestation in normal pregnancy [149] (OR = 4.3, p<0.001) (Table 4). This supports our prediction that proteins that change with gestation in normal pregnancy could be helpful in understanding obstetrical complications and may serve as biomarkers for the prediction of these disorders.

### Biological processes perturbed in late-onset preeclampsia

Gene ontology analysis of the proteins that changed significantly between the cases and controls was performed for each gestational age interval. Despite the inherent limited power of such analysis (due to few significant proteins at each gestational age interval), we have identified biological processes perturbed in late-onset preeclampsia. These gene ontologies included: *small molecule metabolic process* and *positive regulation of apoptotic process* at 8–16 weeks, and *positive regulation of vascular endothelial growth factor receptor signaling pathway*, *positive regulation of cell adhesion*, and *extracellular matrix organization* at 16–22 weeks (OR = 3.1–38.1, all q<0.1) (Table 5).

**Table 5:** Gene ontology (GO) biological processes associated with protein abundance changes with late-onset preeclampsia.

GA Interval	Name	N	Proteins	OR	p	q
8–16	small molecule metabolic process	5	BMP-1;Glutathione S-transferase Pi;Phosphoglycerate mutase 1;RAN;COX-2	5.8	0.007	0.01
8–16	positive regulation of apoptotic process	3	PPID;HMG-1;COX-2	8.1	0.012	0.01
16–22	positive regulation of vascular endothelial growth factor receptor signaling pathway	3	gpIIbIIIa;IL-1b;VEGF121	38.1	0.000	0.00
16–22	positive regulation of cell adhesion	3	Integrin aVb5;Trypsin 2;VEGF121	14.7	0.002	0.02
16–22	extracellular matrix organization	6	MMP-7;gpIIbIIIa;BMP-1;Integrin aVb5;DMP1;Trypsin 2	4.5	0.006	0.04
16–22	cell migration	3	gpIIbIIIa;Integrin aVb5;NCK1	6.5	0.017	0.07
16–22	innate immune response	7	HMG-1;C4b;PRKACA;NCK1;B7-2;Trypsin 2;DUS3	3.1	0.022	0.07
16–22	positive regulation of transcription from RNA polymerase II promoter	5	HMG-1;CDK8/cyclin C;NCK1;IL-1b;VEGF121	3.7	0.023	0.07
16–22	positive regulation of protein phosphorylation	3	gpIIbIIIa;IL-1b;VEGF121	5.4	0.027	0.07
16–22	negative regulation of transcription from RNA polymerase II promoter	3	HMG-1;PPID;VEGF121	4.8	0.035	0.08
16–22	positive regulation of transcription, DNA-templated	3	RAN;IL-1b;B7-2	4.2	0.047	0.09
16–22	extracellular matrix disassembly	3	MMP-7;BMP-1;Trypsin 2	4.1	0.053	0.09

GA: gestational age, N: number of significant proteins belonging to the GO term; OR: enrichment odds ratios; p: p value, q: false discovery rate adjusted p-value. P-values displayed as 0.00 and 0.000 should be considered <0.01 and <0.001 respectively.

## Discussion

### Principal findings of the study

1) The strongest predictors of late-onset preeclampsia are the elevated abundance of maternal plasma MMP-7 early in gestation (8–22 weeks) and the low maternal plasma abundance of PlGF later in gestation (after 22 weeks); 2) the high abundance of MMP-7, CDK8/cyclin C, PPID, and RAN and the low abundance of HSP70 at 8–28 weeks of gestation were associated with late-onset preeclampsia after adjusting for covariates; 3) biological processes perturbed at 8–16 weeks of gestation in patients destined to develop late-onset preeclampsia included: a) a small-molecule metabolic process and b) a positive regulation of the apoptotic process (at 8–16 weeks), while, at 16–22 weeks, the biological processes included a) the positive regulation of the vascular endothelial growth factor receptor signaling pathway, b) the positive regulation of cell adhesion, and c) the extracellular matrix organization; and 4) from 22.1 weeks of gestation onward, the set of proteins most predictive differed in patients who subsequently developed mild versus severe late-onset preeclampsia.

#### Early-onset vs. late-onset preeclampsia: Two different disorders

The syndrome of preeclampsia has been classified into two major clinical conditions, according to the gestational age at diagnosis and delivery [77–79]. Early-onset preeclampsia is characterized by a high frequency of placental maternal vascular lesions of underperfusion and a small placenta [90–94], abnormal umbilical artery and uterine arteries Doppler velocimetry [99–104], an abnormal angiogenic/anti-angiogenic profile [58,59,72,80,109,111], fetal growth restriction [95–98], and a relatively high rate of thrombocytopenia, elevated liver enzyme abnormalities, and the HELLP syndrome [150]. By contrast, late-onset preeclampsia is thought to result from a mismatch between the nutrient supply by the mother and the metabolic demands of the fetus at the end of pregnancy [77–79]. Typically, the placenta is of normal weight [92]; it is less likely to have maternal vascular lesions of underperfusion than the placenta in early-onset disease [90–93]; fetuses are frequently of appropriate or large birth weight for gestational age [105–108]; and the uterine arteries and umbilical artery Doppler velocimetries are generally within normal range [79]. Late-onset preeclampsia is more likely to occur in obese patients [151–153]. Cardiac output, total vascular resistance, and the morphology of the left ventricle, as determined by echocardiography, are also different in early- and late-onset preeclampsia by 24 weeks of gestation [79].

Because the etiologies of early- and late-onset preeclampsia are different, biomarkers predicting their development are expected to diverge. For example, the concentrations of PlGF and anti-angiogenic factors (sFlt-1 and sEng) are good predictors of early-onset preeclampsia, but not of late-onset disease. We undertook the discovery of biomarkers focusing exclusively on late-onset disease: we and other investigators previously addressed the prediction of early-onset preeclampsia [99,101,119–123].

#### MMP-7, a predictor of late-onset preeclampsia

Elevated abundance of MMP-7 in maternal plasma before 22 weeks of gestation was the strongest predictor of late-onset preeclampsia. This matrix metalloproteinase, also called matrilysin, is involved in the degradation of several types of collagen (III, IV, V, IX, X, XI), proteoglycans, fibronectin, elastin, and casein [154]. It is the smallest MMP that circulates in the blood. The main form is pro-MMP-7, which is enzymatically inactive. MMP-7 is involved in innate immune processes, mainly in the lung and gut, due to its proteolytic activity that activates α-defensins (anti-bacterial peptides able to disrupt bacterial membrane) [154]. Indeed, silencing MMP-7 in mice will result in the inability to activate pro-α-defensins in the gut and a higher susceptibility to intestinal bacterial infections [155]. MMP-7 also has an important role in releasing TNF-α from macrophages; and it is involved in the transepithelial migration of neutrophils by cleaving syndecan-1, the main heparan sulphate proteoglycan on the epithelium. Maternal plasma concentration of TNF-α is elevated in preeclampsia [156,157].

Recent evidence suggests that MMP-7 may play a role in atherosclerotic disease, which has many parallels to preeclampsia [158]. Indeed, the SUMMIT Consortium (surrogate markers for micro- and macrovascular hard endpoint for innovative diabetes tools) reported that circulating MMP-7 concentrations were higher in patients with Type 2 diabetes mellitus, correlated with patients’ age, and were independently associated with the prevalence of cardiovascular disease and the burden of atherosclerosis as well as arterial stiffness and plaque inflammation. Baseline MMP-7 concentrations were elevated in patients who had a coronary event during the study period [158]. Circulating concentrations of MMP-7 are significantly higher in patients with histological unstable atherosclerotic carotid lesions compared to patients with stable lesions [159]. In addition, markedly higher mRNA levels of MMP-7 were found within carotid plaques than in arteries without plaques [160]. MMP-7 within the carotid plaques was primarily localized in macrophages [160,161], and *in vitro* studies showed that combined stimulation of inflammatory mediators (TNF-α), oxidized LDL, and hypoxia markedly increased MMP-7 expression in monocytes [160]. In atherosclerotic plaques, MMP-7 is expressed by lipid-laden macrophages [161], the same cells present in acute atherosis of the spiral arteries, a lesion associated with preeclampsia [162,163]. Thus, MMP-7 may contribute to plaque destabilization in patients with carotid artery stenosis. Abbas et al. [160] reported that MMP-7 concentrations were especially higher if the patients were symptomatic within the prior two months of sampling. Moreover, high plasma concentrations of MMP-7 in these patients were independently associated with total mortality [160].

During pregnancy, MMP-7 is expressed in the decidua and trophoblast. In the first trimester, uterine NK cells and macrophages abundant in the decidua express MMP-7; and matrilysin may have a role in the process of transformation of the spiral arteries, because 50%-75% of leukocytes infiltrating and remodeling the vessels are positive for MMP-7 and MMP-9 [164]. During normal pregnancy there is constant expression of MMP-7 in the intermediate trophoblast and decidual cells throughout gestation [165].

Matrilysin is associated with pregnancy complications: 1) its amniotic fluid concentrations are elevated in women with preterm labor and intact membranes who deliver preterm regardless of the presence of intra-amniotic infection [166]; 2) in the placentas of patients with severe preeclampsia, there is extensive immunostaining of all layers of villous trophoblast for MMP-7 [165]; 3) by contrast, placentas from patients with severe early-onset preeclampsia with fetal growth restriction, the interstitial trophoblast cell expression of MMP-3 and MMP-7 are markedly reduced [167]. The authors attributed this finding to the fact that decidual NK cells aggregated near the spiral arteries secrete leukemia inhibitory factor (LIF) that suppresses the expression of MMPs. This may impede the physiological transformation of the spiral arteries, which has been implicated in the pathophysiology of early-onset preeclampsia [167]. Collectively, these reports suggest that MMP-7 may be involved in two fundamental processes associated with the development of preeclampsia: placentation and inflammation.

A previous study reported that MMP-2 is elevated in maternal urine as early as 12–16 weeks of gestation, and an elevated concentration of MMP-2 at 12 weeks predicted the development of preeclampsia with a sensitivity of 100% and a specificity 62.5%; at 16 weeks of gestation, an elevated MMP-2 in maternal urine predicted the development of preeclampsia with a sensitivity of 87.5% and a specificity of 74.1% [168]. The urine concentration of MMP-7 in patients who subsequently developed preeclampsia did not differ from those with a normal pregnancy, but the study could not differentiate between patients who subsequently developed early- and late-onset preeclampsia [168].

#### What are the differences in the proteomic profile between patients with mild and severe late-onset preeclampsia?

The severity of preeclampsia has major implications for maternal and neonatal outcomes. Patients with a mild disease need only timely delivery and observation. By contrast, women with severe preeclampsia have a high rate of maternal morbidity, including eclampsia, abruption, elevated liver enzymes, and emergency cesarean delivery [20]. Therefore, early identification of women who will subsequently develop severe preeclampsia is important as they may benefit from a timely delivery prior to the onset of the severe preeclampsia [169].

Until 22 weeks of gestation, MMP-7 was the most predictive protein for the development of late-onset preeclampsia, either in mild or severe form. After 22 weeks, we observed differences in the set of proteins most predictive of mild or severe preeclampsia. PlGF optimally identified patients destined to develop severe preeclampsia at 22.1–28 and 32.1–36 weeks of gestation, whereas patients destined to develop mild preeclampsia were better predicted by a different set of proteins at each gestational age. These proteins are involved in angiogenesis (e.g., PlGF), coagulation (e.g., tissue factor), cell division (e.g., RAs-related Nuclear protein), and cell-to-cell interaction (e.g., tyrosine-protein kinase Fer). The finding that, after 22 weeks of gestation, PlGF is the best predictor of late-onset preeclampsia, especially in its more severe form, is consistent with previous reports [61,115,170–173]. We and others [55,70,174] presented the use of this angiogenic factor as a tool for the assessment of the impending risk for preeclampsia, demonstrating lower concentrations of PlGF in cases when compared to controls as early as at least six weeks prior to the onset of the disease [61]. Moreover, the determination of this angiogenic factor has prognostic value in patients presenting to the obstetrical triage area with suspected preeclampsia for the identification of those requiring delivery due to impending preeclampsia[171, 172].

#### Identification of patients who subsequently developed late-onset preeclampsia may warrant a two-stage assessment approach

The comparison of the proteomic prediction models built to predict subsets of cases based on the severity of this syndrome suggests that we may need a two-step approach for the prediction of late-onset preeclampsia. Similar to the current paradigm for the identification of patients at risk of aneuploidy, for which a two-step model has been used (the first at 11–13 weeks of gestation includes nuchal translucency and biochemical markers such as hCG and PAPP-A; and the second at 17 weeks includes alpha feto-protein, hCG, and E3, as well as inhibin in cases of quad test) to generate an integrated risk that serves as the basis for further diagnostic tests, e.g., amniocentesis to diagnose aneuploidy [175–178]. Unlike the detection of patients at risk for early-onset preeclampsia, in which maternal background characteristics, PlGF concentration, and maternal blood pressure at the time of sample collection can identify the majority of patients at risk for the development of this syndrome [82,114,118,119], our study indicates that optimal prediction of late-onset preeclampsia may involve two diagnostic steps: the first assessment during early gestation (8–22.1 weeks), using MMP-7, and the second one later during the third trimester (28.1–32 weeks). Until 22.1 weeks, MMP-7 has the highest predictive performance for the identification of patients at risk to develop late-onset preeclampsia regrades to severity, whereas, after 22 weeks, the set of optimal proteomic predictors differs according to the severity of late-onset preeclampsia. This has implications on clinical management, since those who are at risk for the development of severe late-onset preeclampsia may benefit from timely delivery near 37 weeks of gestation, while those who are destined to have a mild disease may continue pregnancy to term under close surveillance.

#### Strengths and limitations

The major strengths of this study are the large number of proteins tested, as well as its longitudinal design and the number of samples included in the analysis, especially during early stages of pregnancy. This is the first study to demonstrate that proteomic profiles identify patients destined to develop severe or mild late-onset preeclampsia as early as 16 weeks with a sensitivity that surpasses that of PlGF. Our study includes mainly African American women; this may limit the generalizability of our results to this ethnic group, which is at much higher risk to develop preeclampsia than other ethnic groups.

It is common in the field of high-dimensional biology to combine predictors (e.g., mRNAs, proteins, metabolites, etc.) in a logistic regression (or other type of prediction model) and report one set of predictive performance indices on the full set of patients used to select the predictors and fit models for this purpose. However, such approaches would lead to optimistically biased performance indices due to at least two sources of bias. The most important is the feature selection bias, since, when selecting from a large pool of candidate biomarkers, it is generally possible to find a few “biomarkers” that appear to predict the outcome better than expected by chance (e.g., AUC>0.5). The second source of bias comes from tuning (estimating) the weights (co-efficients) of a predefined set of predictors to fit the available data. We avoided these common pitfalls by relying on bootstrap-estimated performance indices. With this procedure, predictor/feature selection and model fitting are repeated 100 times on data from a training set of patients while the model is tested on data from patients left out at each iteration. As shown in Fig 1, the LOOCV AUC and sensitivity estimates of the best combination of markers are in the worst case as low as the one of PlGF, yet we only claim better prediction compared to PlGF alone in the first two intervals when the bootstrap-based estimates of multi-marker models are significantly higher than those of PlGF.

Also, we and others [143] have addressed the problem that indicates when high-dimensional data are used to build prediction models, the same prediction performance can be achieved with widely different sets of predictors, due, among other reasons, to the correlation that may exist among them. Therefore, instead of emphasizing the sets of proteins identified in the final models (Table 2), we focused our inferences on the proteins that appear to be selected as the best predictors more often during the 100 different bootstrap iterations. For instance, while PlGF was the most reliable predictor of late-onset preeclampsia in the interval 22.1–28 weeks, being included in the best combination 24/100 times, when all data was used to fit the final model, RAN and METAP1 appeared to be the best choices even though they were selected 12 and 16 times in the best combination out of 100 bootstrap trials.

## Conclusion

We present herein new biomarkers to identify patients who will develop late-onset preeclampsia based on a high through-put proteomics method. We report that elevated MMP-7 early in gestation (8–22 weeks) and low PlGF later in gestation (after 22 weeks) are the strongest predictors for the subsequent development of late-onset preeclampsia, hence suggesting that the optimal identification of patients at risk may involve a two-step diagnostic approach. In addition, abnormal proteomic profiles before 22 weeks of gestation are associated with perturbation of several biological processes including the positive regulation of vascular endothelial growth factor receptor signaling pathway.

## Supporting information

S1 File Proteomics data used in the analyses presented in this study. Protein abundance data for each sample (rows) and each of the 1125 proteins is given in this table. ID: anonymized identifier indicator of the patient, GA: gestational age at sample, LatePE: is 1 for late preeclampsia and 0 for normal pregnancy. Protein symbol and names provide by Somalogic, Inc, are included above the protein accession numbers.
(CSV)

S2 File Summary of differential abundance analysis between late-onset preeclampsia and normal pregnancy in five intervals of gestation. Thirty-six proteins that were significant (Sig.) (q<0.25 and fold change >1.1) in at least one interval are shown. Adjustment was performed for BMI, maternal age, parity and smoking. FC: linear fold change, with negative values denoting lower while positive values denoting higher level in cases than in controls, p: p-value, q: adjusted p-value, GA: gestational age. The column labeled as “Changes with GA” indicates whether the protein abundance changes with gestational age [128].
(XLS)
